# Paediatric Nonfunctioning Adrenocortical Carcinoma with Extension up to Right-Side Heart: Cardiac Surgery Approach

**DOI:** 10.1155/2016/2321017

**Published:** 2016-07-14

**Authors:** Federica Iezzi, Andrea Quarti, Chiara Surace, Marco Pozzi

**Affiliations:** Department of Paediatric and Congenital Cardiac Surgery and Cardiology, Azienda Ospedaliero-Universitaria Ospedali Riuniti Ancona “Umberto I, G. M. Lancisi, G. Salesi” Ancona, Via Conca 71, 60128 Ancona, Italy

## Abstract

Adrenocortical carcinoma is a rare malignancy. Due to late diagnosis and no adequate effective adjuvant treatment, prognosis remains poor. Only approximately 30% of these malignancies are confined to the adrenal gland when they are diagnosed, as these tumors tend to be found years after their genesis. Cardiac involvement of adrenal carcinoma is very rare. We report a rare case of a 7-year-old female with right adrenal cortical carcinoma, involving the right-side heart.

## 1. Introduction

Childhood adrenocortical carcinoma is a rare and highly malignant neoplasm, with propensity to involve adrenal vein, renal vein, and inferior vena cava, rarely reaching up to right atrium. Dissemination of the tumor occurs in 82% of the patients with a median survival of 14.5 months. Patients with nonfunctioning tumor usually have no symptoms [1].

The management of choice for adrenal tumors with thrombotic extension into inferior vena cava is* en bloc* tumor removal. Accurate preoperative radiographic evaluation of primary tumor and determination of any venous tumor involvement are therefore absolutely critical to surgical approach and successful management.

## 2. Case Report

A 7-year-old child was referred to our department, as urgent case, from another hospital due to echocardiography evidence of a huge mass in inferior vena cava, reaching right heart, up to right ventricle outflow tract.

The physical examination revealed a sistodiastolic murmur on the third intercostal space of left sternal border.

Pulmonary and abdominal examination was unremarkable. She complained of occasional and aspecific abdominal pain.

The laboratory findings included elevated levels of lactate dehydrogenase (507 U/L) and neuron-specific enolase (86 ng/mL).

Transthoracic echocardiography showed a round, long, mobile, nonobstructing mass invading inferior vena cava wall, extending into right atrium, prolapsing through tricuspid valve, and reaching the outflow tract of right ventricle. The isoechogenic intracardiac mass (5,3 cm × 1 cm), stipple in texture structure with well-demarcated borders, was adherent to inferior vena cava wall, with its incomplete obstruction and consequent dilation of hepatic veins. There was no evidence of right ventricular outflow tract obstruction. No other intracardiac masses were noted, and the tricuspid valve was uninvolved, with normal color-flow and spectral Doppler evaluation.

**Figure 1:**
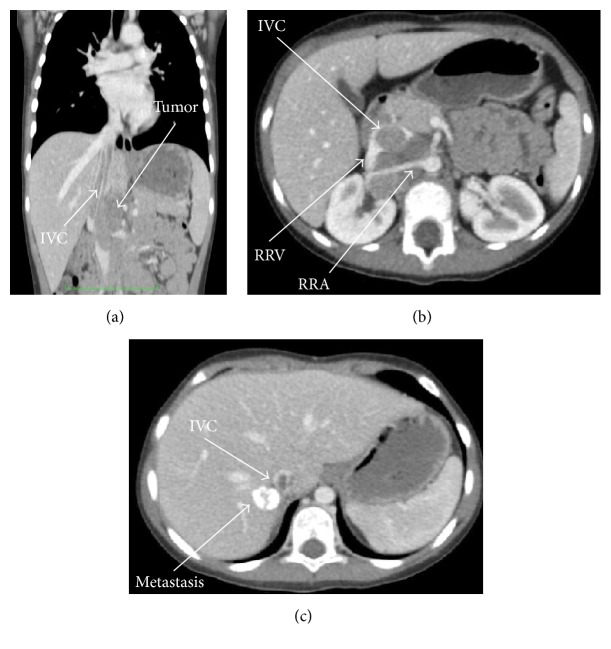
Computed tomographic scanning showing (a) incomplete obstruction of inferior vena cava (IVC) by tumor mass (b) incorporating the right renal artery (RRA) and the right renal vein (RRV). Abdominal imaging revealed a calcified solitary hepatic lesion (c).

Enhanced computed tomographic scanning revealed a 5 cm × 4,5 cm large mass in right suprarenal location with infiltration into right renal artery, superior mesenteric artery, right renal vein, distal tract of left renal vein, and inferior vena cava, reaching up to right atrium. Abdominal imaging revealed also a solitary 26 mm hepatic lesion in segment V (Figure 1).

The exam pointed to a diagnosis of adrenal carcinoma with extension of tumor into right heart.

Due to the risk of embolism from the tumor, continuous infusion of heparin was immediately initiated. After multidisciplinary meeting with paediatric cardiothoracic surgeons, paediatric cardiologists, oncologists, vascular surgeons, and general paediatric surgeons, due to the serious risk of fatal pulmonary embolization of the large atrioventricular mass, the patient underwent emergency cardiac surgery.

Cardiopulmonary bypass was instituted by cannulation of ascending aorta and by use of a single venous cannula in superior vena cava, direct through right atrium. Circulatory arrest at 22°C was employed together with cardioplegic arrest. The tumor was found to have a wide attachment into the inferior vena cava wall. Through a right atriotomy the excision of tumor mass with removal of intracaval and right heart extension was performed. The cardiac postoperative course was uneventful. Short-interval follow-up showed no tumor or thrombus protruding into right heart.

Extemporaneous histopathology of the specimen revealed cellular pleomorphism with large nuclei, abundant cytoplasm, and numerous small vascular channels which were findings compatible with an endocrine tumor.

**Figure 2:**
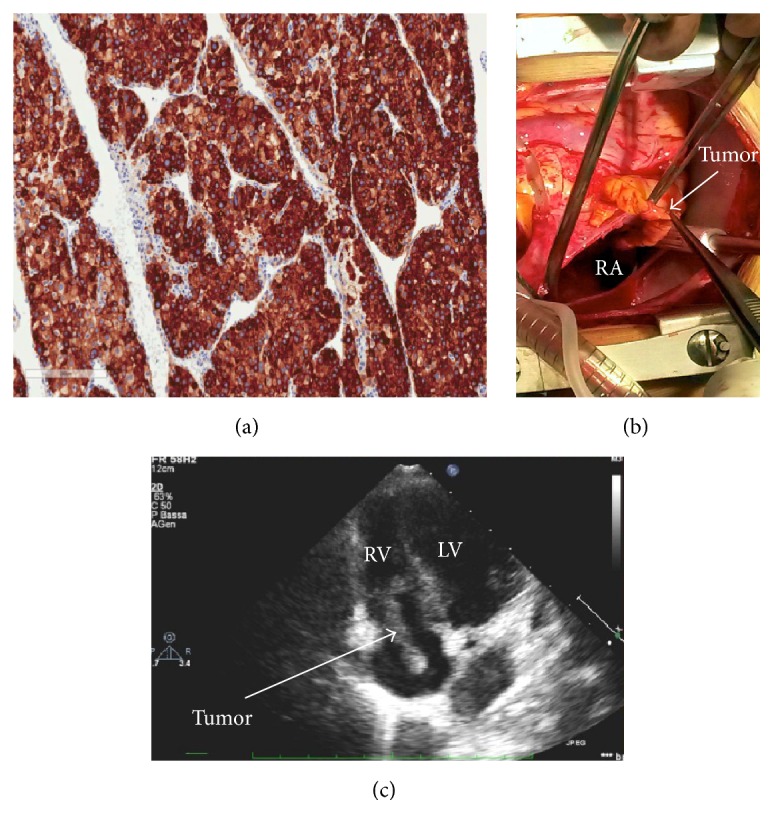
Histopathological examination showing high mitotic activity. Immunoreactivity for the adrenal cortical antigens inhibin (a). Transthoracic echocardiographic (c) findings in the apical four-chamber view showing the serpentine mass extending from the inferior vena cava to the right atrium, through the tricuspid valve to the right ventricle (RV). Intraoperative view (b) showing the tumor after opening of the right atrium (RA).

Histopathological examination of the right atrial mass revealed malignant adrenal epithelial cells with nuclear pleomorphism, high mitotic activity (mitotic rate 35 per 50 high power fields), and confluent areas of necrosis. Barely visible fibrous bands separated the tumor mass into fine lobules. A small area of the tumor presented a broad-trabecular pattern and was predominantly composed of clear-cytoplasm cells. Overall, clear cells made up no more than 25% of the whole tumor area (Figure 2).

The tumor cells showed strong diffused immunoreactivity for vimentin, synaptophysin, and inhibin. Melan A expression was diffusely positive. The tumor cells were calretinin, chromogranin, S100, cytokeratin AE1/AE3, and CAM 5.2 negative.

## 3. Discussion

In the present case transthoracic echocardiography identified the cardiac involvement of an adrenocortical carcinoma. Tumors that affect the right-side heart include primary neoplasms. For the high risk of right ventricle outflow tract obstruction and pulmonary embolism heart surgery approach was mandatory [2].

Inferior vena caval invasion is well-known complication as well, more commonly seen with right-sided heart masses. The prognostic value of intravenous extension is still controversial and debated but does not represent a contraindication to surgery. Involvement of the inferior vena cava was reported in only single case reports and small series, in which complete surgical resection remains the most effective treatment [3].

The appropriate treatment for adrenocortical carcinoma is not well established, and the effectiveness of other modalities, such as chemotherapy and radiotherapy, is not proven.

Neoadjuvant therapy with systemic chemotherapy and mitotane adrenolytic strategy should be considered for patients with primarily incomplete resectable, inoperable, or disseminated adrenocortical carcinoma and tumor spillage.

Mitotane and chemotherapy with etoposide, doxorubicin, and cisplatin plus mitotane are the only therapies with demonstrated efficacy in advanced adrenocortical carcinoma. Prognostic and predictive factors are needed to identify patients who could obtain the best benefit from these treatments. Despite the strong rationale for their use, clinical trials on molecular targeted therapies have failed to demonstrate that these drugs are efficacious in the management of this extremely rare disease. Mitotane and cytotoxic chemotherapy are currently the only systemic treatments available for locally advanced or metastatic adrenocortical carcinoma not amenable to surgery.

Currently our patient is following systemic chemotherapy plan, in preparation for oncological surgery.

In light of these findings, surgery remains the best treatment modality for adrenocortical carcinoma in the early stages. However, data regarding the effectiveness of surgery in the management of metastatic tumor remain scarce [4].

Improved survival is seen in patients who present at an early stage and have complete primary resection. Five-year survival for patients with stage IV tumors is usually less than 20%.

Detection of local invasion or tumor extension into the inferior vena cava, as well as lymph node or other metastases (lung and liver), is important for planning surgery.

In the case of inoperable local infiltrating or metastatic adrenocortical carcinoma, surgical excision of the primary tumor or metastasis should be considered in case of documented response after neoadjuvant chemotherapy, when a radical resection seems to be feasible [5].

## 4. Conclusions

Despite a comprehensive biochemical, radiologic, and histologic assessment of adrenocortical carcinoma, planning an operative procedure with a heart involvement remains a challenge.

Due to the tendency of the tumor to disseminate, a precise preoperative assessment is essential.
